# Thermodynamic Study of Formamidinium Lead Iodide (CH_5_N_2_PbI_3_) from 5 to 357 K

**DOI:** 10.3390/e24020145

**Published:** 2022-01-18

**Authors:** Andrea Ciccioli, Alessandro Latini, Alessio Luongo, Natalia N. Smirnova, Alexey V. Markin, Stefano Vecchio Ciprioti

**Affiliations:** 1 Department of Chemistry, Sapienza University of Rome, P.le A. Moro 5, Building CU014, I-00185 Rome, Italy; andrea.ciccioli@uniroma1.it (A.C.); alessandro.latini@uniroma1.it (A.L.); alessio.luongo@uniroma1.it (A.L.); 2 Department of Chemistry, National Research Lobachevsky State University of Nizhny Novgorod, 23/5 Gagarin Av., 603950 Nizhny Novgorod, Russia; smirnova@ichem.unn.ru; 3 Department of Basic and Applied Science for Engineering (S.B.A.I.), Sapienza University of Rome, Via del Castro Laurenziano 7, Building RM017, I-00161 Rome, Italy

**Keywords:** formamidinium lead iodide, adiabatic calorimetry, heat capacity, standard thermodynamic functions

## Abstract

In the present study, the molar heat capacity of solid formamidinium lead iodide (CH_5_N_2_PbI_3_) was measured over the temperature range from 5 to 357 K using a precise automated adiabatic calorimeter. In the above temperature interval, three distinct phase transitions were found in ranges from 49 to 56 K, from 110 to 178 K, and from 264 to 277 K. The standard thermodynamic functions of the studied perovskite, namely the heat capacity *C*°_p_(*T*), enthalpy [*H*^0^(*T*) − *H*^0^(0)], entropy *S*^0^(*T*), and [*G*°(*T*) − *H*°(0)]/*T*, were calculated for the temperature range from 0 to 345 K based on the experimental data. Herein, the results are discussed and compared with those available in the literature as measured by nonclassical methods.

## 1. Introduction

Since their first appearance in 2009, perovskite solar cells have attracted a great deal of attention, owing to their relatively simple technology and good performance. Nowadays, they constitute the photovoltaic technology with the fastest-growing conversion efficiency [1,2]. The first compound of the hybrid perovskite family to be extensively studied for photovoltaic devices was methylammonium lead iodide, CH_3_NH_3_PbI_3_ [3,4,5]. Its intriguing photophysical properties, such as its direct band gap, with a value very near optimal one for photovoltaic conversion of solar radiation, and its defect tolerance, were thoroughly studied following the discovery of its exceptional photovoltaic performance [6,7,8]. However, the limited chemical and thermal stability of CH_3_NH_3_PbI_3_ immediately emerged as a very serious problem. In spite of their impressive performances in photovoltaic devices, perovskite solar cells seem quite far from commercial debut. 

The search for alternative compounds with enhanced stability led researchers to focus on cesium lead iodide (CsPbI_3_) and formamidinium lead iodide (CH_5_N_2_PbI_3_, FAPI) as the most promising options [9]. Both compounds have been extensively tested, either pure and in solid solutions (also with CH_3_NH_3_PbI_3_) [10,11,12,13]. Both CsPbI_3_ and CH_5_N_2_PbI_3_ occur as black phases (useful for photovoltaic purposes) at relatively high temperatures (*T* > 320 °C for CsPbI_3_ [14] and *T* > 185 °C for CH_5_N_2_PbI_3_ [15,16]) and yellow phases at lower temperatures. 

In order to assess the stability under various operating conditions, a full thermodynamic characterization of the material and of its possible decomposition pathways is mandatory. To date, enthalpy and free-energy data have been published for CH_3_NH_3_PbI_3_ [17,18,19,20,21,22] and, to a lesser extent, for CsPbI_3_ [23,24,25,26]. Recently, data on the thermodynamic stability of CH_5_N_2_PbI_3_ were published by our group [27]. 

In this connection, the measurement of heat capacities from low temperature up to decomposition temperatures is of utmost importance to derive absolute entropy values and to calculate the values of thermodynamic quantities at temperatures different from those explored in the experiments. Furthermore, the study of heat capacities is of great help to investigate the low-temperature phase transitions that occur in the material, the dynamics of molecular motions, and the nature of the molecule–cage interaction [28], which is important to clarify the role of the organic cations in the photovoltaic performance. To the best of our knowledge, heat-capacity values measured by adiabatic calorimetry are available in literature only for CH_3_NH_3_PbI_3_ [5,29]. In regard to CH_5_N_2_PbI_3_, few papers are available wherein the heat capacity was reported [28,30,31]. In particular, Fabini and colleagues measured the heat capacities of powder samples in temperature ranges across the phase transitions by the pulse-relaxation method [28] and by differential scanning calorimetry [30], whereas Kawachi and colleagues [31] reported measurements of single crystal samples by the relaxation technique. The aim of the present paper is to present the first experimental determination of the heat capacities of CH_5_N_2_PbI_3_ in the temperature range from 5 to 357 K by classic adiabatic calorimetry and to provide the thermodynamic functions derived therefrom.

## 2. Materials and Methods

Synthesis and structural characterization of either methylammonium lead iodide (MAPI) or FAPI were carried out according to procedures reported in detail in previous studies [18,27]. 

The heat capacity of MAPI and FAPI was measured over the range of *T* = (5–357) K using an automatic BCT-3 low-temperature adiabatic calorimeter. The calorimeter was manufactured at ‘‘Termis” joint-stock company at the All-Russian Metrology Research Institute, Moscow, Russia. Its design and operation procedure are described in [32]. The iron and rhodium thermometer (resistance at *T* = 273.1 K is ~51 Ω) was calibrated on the basis of ITS-90 [33]. Liquid helium and nitrogen were used as cooling agents.

The ampoule with the fine crystalline substance was filled with dry helium as a heat-exchange gas to a pressure of 4 kPa at room temperature. The reliability of the calorimeter was checked by measuring *C*°_p,m_ of standard samples of high-purity copper [34], standard synthetic corundum, *n*-heptane (chromatographically pure) [35], and K-3 benzoic acid [36,37] prepared at the Institute of Metrology of the State Standard Committee of the Russian Federation.

The test of the calorimeter revealed that average deviations of the experimental data from the precision literature data were 2% at *T* = (5–15) K, 0.5% at *T* = (15–40) K, and 0.2% at *T* = (40–357) K. The phase-transition temperatures were measured within a standard uncertainty of about u(*T*) = 0.01 K. The mass of the sample loaded in a 1.5 cm^3^ thin-walled cylindrical titanium ampoule of the BCT-3 device was 1.5551 g. The *C*°_p,m_ measurements were carried out in the range of *T* = (5–357) K.

The experimental *C*°_p,m_ values (Table 1) were obtained in six runs. The heat capacity of the sample varied from 54 to 92% of the total heat capacity of the (calorimetric ampoule + substance) in the range of *T* = (5–357) K.

The experimental *C*°_p,m_ points were smoothed in all the temperature regions for which any transformations were absent, according to the following polynomials (Equations (1)–(3)):

(1) $$C{{}^{\circ}}_{p,m}^{}=\sum_{j=1}^{n}A_{j}\cdot {\left(T/30\right)}^{j}\;\;\;\;(177.35–264.5)\;K\;\mathrm{and}\;(277.0–348.6)\;K$$

(2) $$C{{}^{\circ}}_{p,m}^{}=\sum_{j=1}^{n}A_{j}\cdot \ln{\left(T/30\right)}^{j}\;\;\;\;(15.1–49)\;K\;\mathrm{and}\;(55.3–169.25)\;K$$

(3) $$\ln C{{}^{\circ}}_{p,m}^{}=\sum_{j=1}^{n}A_{j}\cdot \ln{\left(T/30\right)}^{j}\;\;\;\;(5.1–15.59)\;K$$

where *A*_j_ represents the fitting polynomial coefficients, and n is the number of coefficients. The standard atomic masses recommended by the IUPAC Commission in 2013 [38] were used in the calculation of all molar quantities.

**Table 1:** The experimental values of the molar heat capacity of CH_5_N_2_PbI_3_ in J·K^−1^·mol^−1^, M(CH_5_N_2_PbI_3_) = 63,297,507 g·mol^−1^, *p*° = 0.1 MPa.

*T*/K	*C*°_p,m_/ J·K^−1^·mol^−1^	*T*/K	*C*°_p,m_/ J·K^−1^·mol^−1^	*T*/K	*C*°_p,m_/ J·K^−1^·mol^−1^
Series 1					
5.16	2.56	17.06	34.24	51.44	134.6
5.31	2.75	17.56	35.74	52.62	172.6
5.55	3.20	18.05	37.35	53.87	149.8
5.77	3.52	18.55	38.92	55.27	124.6
5.99	3.96	19.05	40.43	56.61	125.8
6.24	4.30	19.55	42.09	57.89	126.6
6.53	4.84	20.05	43.76	59.16	127.9
6.83	5.43	20.89	46.08	60.44	128.6
7.13	6.04	22.04	49.71	61.72	129.6
7.44	6.61	23.21	53.26	62.99	130.7
7.75	7.32	24.38	56.79	64.27	131.3
8.07	8.00	25.57	59.98	65.54	132.4
8.40	8.72	26.76	63.21	66.82	132.8
8.72	9.51	27.96	66.51	68.10	133.6
9.05	10.3	29.17	69.85	69.38	134.2
9.38	11.1	30.37	73.14	71.10	135.4
9.72	12.0	31.59	76.28	73.26	136.5
10.06	12.9	32.82	79.41	75.42	137.6
10.45	13.9	34.04	82.20	77.57	139.0
10.90	15.2	35.27	85.00	79.74	140.3
11.35	16.5	36.50	87.29	81.90	141.5
11.81	17.9	37.74	89.90	84.06	142.9
12.27	19.2	38.98	92.47	86.23	144.2
12.73	20.8	40.23	94.76	88.40	145.7
13.20	22.2	41.48	97.28	90.57	147.1
13.67	23.7	42.73	100.2	92.74	147.9
14.14	25.1	43.97	103.3	94.91	149.3
14.62	26.6	45.22	105.8	97.08	150.4
15.11	27.97	46.47	108.3	99.26	151.9
15.59	29.58	47.73	110.4	101.63	153.3
16.08	31.09	48.98	115.6	104.22	154.2
16.57	32.75	50.21	128.0		
Series 2					
44.57	104.0	49.05	114.7	52.90	176.5
45.42	105.8	49.93	124.5	54.48	127.4
46.33	107.8	50.84	129.8	55.51	123.9
47.23	110.1	51.74	138.4	57.70	126.9
48.13	112.3	52.50	171.7	59.21	128.2
Series 3					
84.76	143.3	156.2	200.20	224.31	175.8
86.66	144.8	158.8	204.09	226.91	175.9
88.35	145.7	161.4	208.57	229.51	177.0
90.04	146.5	164.0	213.7	232.12	177.2
91.72	147.6	166.6	219.2	234.75	177.2
93.41	147.8	169.2	226.3	237.38	178.6
95.08	149.5	171.9	227.4	240.00	178.9
96.78	150.2	174.6	173.0	242.61	180.0
99.54	151.6	177.4	169.6	245.22	180.6
102.49	153.5	180.0	169.6	247.86	180.9
105.07	155.0	182.6	169.4	250.48	181.6
107.65	156.5	185.3	169.3	253.10	182.1
110.23	158.0	187.9	169.6	255.72	182.8
112.81	159.9	190.5	169.9	258.34	183.5
115.40	161.7	193.1	170.2	260.97	184.6
118.00	163.2	195.7	170.5	263.62	185.4
120.57	165.1	198.3	170.8	266.26	186.6
131.30	174.1	200.9	171.6	268.88	188.4
134.33	176.2	203.5	171.9	271.50	194.0
137.46	179.6	206.1	172.3	274.12	194.3
140.49	182.8	208.7	172.8	276.77	188.6
143.09	185.9	211.3	173.3	279.42	186.6
145.70	188.0	213.9	173.7	282.06	186.9
148.31	190.4	216.5	174.2	284.70	187.2
150.93	192.9	219.1	174.4	287.34	187.0
153.54	196.7	221.7	175.3	289.97	188.3
Series 4					
173.77	183.3	220.70	174.9	277.37	186.6
177.43	169.3	223.74	175.5	280.60	185.9
180.54	169.2	226.84	176.3	283.83	186.1
183.62	169.5	229.92	177.2	287.06	186.4
186.70	169.6	233.04	177.5	291.10	187.2
189.78	169.8	236.18	178.5	293.54	187.2
192.86	169.7	239.31	179.2	296.79	187.6
195.93	170.2	242.43	180.0	300.04	187.8
199.01	170.8	245.56	180.2	303.93	187.9
202.08	171.5	248.72	180.8	308.43	188.3
205.16	172.0	251.86	181.6	313.84	189.4
208.24	172.5	255.01	182.2	318.91	190.1
211.33	172.8	258.17	183.2	323.13	190.9
214.42	173.3	261.33	184.0	327.41	191.1
217.51	174.2	264.50	185.3	331.66	191.7
220.60	174.2	267.68	187.5	335.91	192.2
223.74	175.5	270.85	194.3	340.15	191.8
217.51	174.2	274.14	191.9	344.39	194.0
Series 5					
119.77	164.5	185.93	171.6	265.82	192.5
122.18	166.2	188.99	172.2	268.91	196.1
124.25	167.2	192.06	172.1	272.00	201.5
126.31	169.6	195.12	172.8	275.12	195.2
128.36	174.8	198.18	173.2	278.26	193.5
130.40	179.3	201.23	174.0	281.39	194.3
132.45	178.1	204.31	174.5	284.53	191.5
134.52	178.8	207.37	174.9	287.67	191.1
136.57	183.6	210.43	175.4	290.83	188.6
138.60	186.4	213.49	176.1	294.00	188.3
140.59	187.6	216.55	176.8	297.17	187.8
142.78	182.6	219.61	177.3	300.35	188.4
145.02	182.7	222.67	178.4	304.13	189.5
147.19	184.0	225.73	179.3	308.31	189.2
149.36	185.3	228.79	180.8	312.49	189.7
151.53	187.3	231.86	181.2	316.67	190.1
154.68	190.3	234.94	182.6	320.87	190.6
158.23	193.7	238.02	183.1	325.07	190.7
161.27	197.3	241.09	183.4	329.26	191.6
164.32	201.5	244.16	183.8	333.45	191.8
167.36	205.8	247.25	185.0	337.63	192.8
170.41	211.8	250.33	186.0	341.80	194.8
173.53	184.3	253.45	187.4	345.97	194.8
176.70	171.0	256.54	187.7	350.08	194.5
179.80	171.4	259.63	189.2		
182.87	171.4	262.72	190.7		
Series 6					
101.53	151.9	179.46	171.5	258.04	190.5
104.98	155.4	183.83	172.0	262.44	192.5
107.99	157.1	188.18	172.7	266.85	194.5
110.99	159.4	192.54	173.0	271.24	202.5
116.70	164.2	196.88	174.0	275.67	196.4
121.66	168.5	201.23	174.9	280.14	195.9
125.96	172.1	205.58	175.7	284.60	192.8
130.22	181.9	209.93	176.7	289.17	191.5
134.52	182.5	214.28	177.2	293.67	189.0
138.82	188.7	218.66	178.8	298.18	188.9
143.13	190.3	223.02	179.8	303.49	190.1
148.24	183.8	227.37	181.9	309.40	189.9
153.27	186.7	231.71	183.1	315.33	190.1
157.59	190.9	236.09	184.9	321.27	190.6
161.91	195.5	240.48	185.3	327.22	190.9
166.22	200.9	244.86	187.2	333.16	192.4
170.53	207.8	249.26	187.2	339.11	194.5
174.98	174.2	253.65	188.9	345.01	195.2

## 3. Results and Discussion

### 3.1. Heat Capacity

A preliminary set of heat-capacity measurements under the identical operative conditions used for the tested compound were carried out on MAPI in order to check the internal consistency of either adiabatic measurements. The data of three experimental runs for MAPI are compared with the available literature data in Figure S1 [5]. A good agreement was found with relative deviations that do not exceed 0.9% up to—250 K and 3% in the range of 250–357 K, thus confirming that a reliable *C*°_p,m_ may also be expected for FAPI.

**Figure 1:**
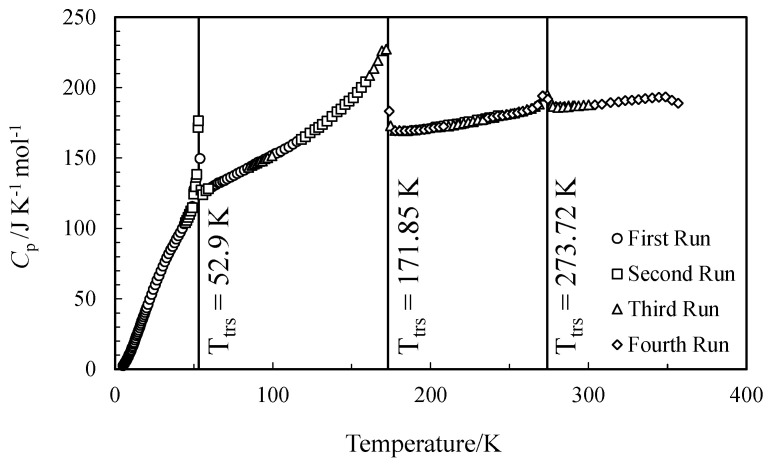
Molar heat capacities of formamidinium lead iodide (FAPI) in the range of 5–357 K.

The experimental values of the molar heat capacity of FAPI in the range of 5–357 K and the smoothing plot, *C*°_p,m_ = *f*(*T*), are illustrated in Table 1 and Figure 1, respectively. 

The *C*°_p,m_ values were smoothed according to Equations (1)–(3) using a polynomial-regression least-square method, while the corresponding fitting coefficients are listed in Table 2.

**Figure 2:**
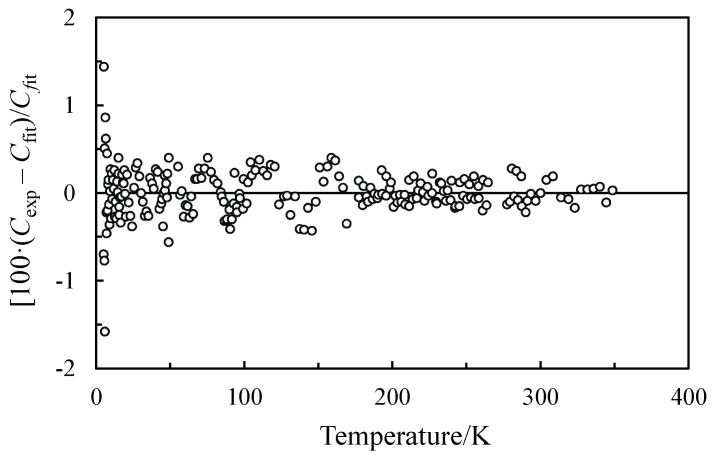
Percentages of deviation of the experimental heat capacity of FAPI from the fitting values.

The relative deviation of the experimental data from the fitting values related to the studied compound is illustrated in Figure 2.

Three distinct phase transitions were found in the ranges of 49–56 K, 110–178 K, and 264–277 K. The characteristics of these transitions are summarized in Table 3.

By means of thermal-expansion measurements, the hexagonal δ-FAPI phase was reported to undergo two phase transitions at 54.5 K and 173.0 K [39], in agreement with our findings. A transition at around 50 K was also reported in [28] (heat-capacity measurements of a powder sample by the pulse-relaxation technique) in the form of two closely-spaced peaks and assigned to the glassy freezing of molecular motions. The total entropy change reported in [28], ranging from 1.7 to 2.2 JK^−1^ mol^−1^, is similar to our result (see Table 3). However, this transition was not reported in [31], wherein a crystal sample was used. The anomaly at around 275 K could corresponds to the previously reported λ-shaped continuous tetragonal-to-cubic (β to α) phase transition [31] due to the formation of small amounts of tetragonal phase in our sample.

### 3.2. Standard Thermodynamic Functions

The standard thermodynamic functions of FAPI reported in Table 4 were calculated from the *C*°_p,m_ values in the temperature range of 0–345 K. To calculate the standard thermodynamic functions of FAPI, its *C*°_p,m_ values were extrapolated from 5 to 0 K according to the Debye law and the multifractal theory of heat capacity in the extremely low-temperature limit [40,41,42]:

*C*°_p,m_ = *nD*(Θ_D_/*T*) (4)

where *n* is the number of degrees of freedom, *D* is the Debye function, and Θ_D_ refers to the Debye characteristic temperature. The parameters selected for this study are *n* = 6 and Θ_D_ = 60.5 K. They were selected so that the errors associated with the heat capacity in the region below 20 K did not exceed the experimental error of its determination.

The values of *H*°(*T*) − *H°*(0) and *S*°(*T*) were estimated in the temperature range of 0–345) K by the numerical integration of *C*°_p,m_ = *f*(*T*) and *C*°_p,m_ = *f*(ln*T*) values, respectively. The values of −[*G*°(*T*) − *H*°(0)]/*T* were determined according to Equation (5):

−[*G*°(*T*) − *H*°(0)]/*T* = −[*H*°(*T*) − *H°*(0)]/*T* + *S*°(*T*) (5)

where all details related to the procedure adopted are available in [43].

As mentioned in the Introduction, the occurrence of gas-releasing decomposition reactions is one of the most severe obstacles on the road to the practical application of hybrid perovskites in photovoltaic technology. The absolute entropy of CH_5_N_2_PbI_3_ measured in this work, *S*°(298 K) = 385.5 J·K^−1^·mol^−1^, enables the calculation of the entropy change of the possible decomposition reactions undergone by the compound under real-use conditions.

Various decomposition processes have been identified and proposed in the literature for CH_5_N_2_PbI_3_ based on Knudsen effusion mass spectrometry, thermogravimetry/mass spectrometry, infrared spectroscopy, and gas chromatography/mass spectrometry [27,44,45,46], leading to the formation of volatile products, such as hydrogen iodide, formamidine, ammonia, hydrogen cyanide, and sym-triazine:

CH_5_N_2_PbI_3_ (s) → PbI_2_ (s) + HI (g) + CH_4_N_2_ (g) (6)

CH_5_N_2_PbI_3_ (s) → PbI_2_ (s) + HI (g) + NH_3_ (g) + HCN (g) (7)

CH_5_N_2_PbI_3_ (s) → PbI_2_ (s) + HI (g) + NH_3_ (g) + 1/3 H_3_C_3_N_3_ (g) (8)

The following values of the absolute entropy at 298 K (expressed in J K^−1^ mol^−1^) were retrieved from the literature for the species involved in the above reactions: PbI_2_ (s), 174.85 [47]; HI (g), 206.60 [47]; NH_3_ (g), 192.77 [47]; HCN (g), 201.82 [47]; and H_3_C_3_N_3_ (g), 271.6 [48]. The value of S°(298) for formamidine, CH_4_N_2_ (g), is not apparently available, but an estimate can be obtained from the entropy change of the dissociation reaction of CH_4_N_2_ (g) → NH_3_ (g) + HCN (g), which was evaluated as 146.5 J K^−1^ mol^−1^ by ab initio calculations [49]. This leads to a *S*°(298) value of 248.1 J K^−1^ mol^−1^ for CH_4_N_2_ (g). Using the above values, the entropy changes of the above reported reactions are (in J K^−1^ mol^−1^): Δ*S*°(298) (6) = 244.1, Δ*S*°(298) (7) = 390.5, Δ*S*°(298) (8) =279.3. In conjunction with the corresponding enthalpy changes, these values can be of help for the prediction of the thermodynamic stability of CH_5_N_2_PbI_3_ as a function of temperature for the various decomposition channels.

## 4. Conclusions

This study reports original results regarding the calorimetric study on formamidinium lead iodide (FAPI). In particular, the heat capacity of FAPI was measured in the experimental temperature range of 5–357 K by precise vacuum adiabatic calorimetry. In the lower temperature range, two phase transitions were observed between 50 and 55 K and 110 and 178 K. A C°_p,m_ anomaly was also found at around 274 K. A good agreement was found with data available in the literature (determined by unconventional methods). By numerical integration of the fitted *C*°_p,m_ and *C*°_p,m_/*T* values, the standard enthalpy [*H*°(T) − *H*°(0)] the entropy *S*°(*T*), and −[*G*°(*T*) − *H*°(0)]/*T* values were determined over the temperature range of 0–345 K.

## Figures and Tables

**Table 2:** The polynomial-fitting coefficients of the temperature dependence of the molar heat capacity of CH_5_N_2_PbI_3_.

Δ*T*/K	5.1–15.59	15.1–49	55.3–169.25	177.35–264.5	277–348.6
Polynomial Equation	Equation (3)	Equation (2)	Equation (2)	Equation (1)	Equation (1)
Polynomial coefficients *A*_j_/J·K^−1^·mol^−1^					
*A*_1_	6.98063245569	72.1805440502	665.255636971	45681.4280583	8877.64561611
*A*_2_	23.7955920199	77.9940374434	−3441.21621166	−37665.2837805	−4276.71076903
*A*_3_	74.7572736802	4.92397788153	8727.22957825	12968.2759007	846.025046732
*A*_4_	129.714906491	−42.6554451220	−11401.7231003	−2377.65802029	−84.1041302983
*A*_5_	127.775856795	14.2864214298	8200.53002955	244.791040664	4.19903809680
*A*_6_	72.6841227853	168.566960506	−3085.56203393	−13.4148724548	−0.0841152839781
*A*_7_	22.2866932511	179.845822583	476.985049821	0.305655340534	
*A*_8_	2.85860464508	42.4593828923			

**Table 3:** The characteristics of transitions for CH_5_N_2_PbI_3_.

Transition	Δ*T*/K	*T*_max_/K	*C*°_p,m_/J·K^−1^·mol^−1^	Enthalpy/J·mol^−1^	Entropy/J·K^−1^·mol^−1^
I	49.5–55.5	52.9	176.5	132.5	2.5
II	110.0–177.5	171.85	227.4	1569	10.3
III	264.5–277.4	273.72	195.3	56.6	0.21

**Table 4:** Thermodynamic functions of CH_5_N_2_PbI_3_. *M*(CH_5_N_2_PbI_3_) = 632.97507 g·mol^−1^. *p*° = 0.1 MPa.

*T*/K	*C*°_p,m_/ J·K^−1^·mol^−1^	[*H*°(*T*) − *H*°(0)]/ kJ·mol^−1^	*S*°(*T*)/ J·K^−1^·mol^−1^	−[*G*°(*T*) − *H*°(0)]/*T* J·K^−1^·mol^−1^
Crystal III				
5	2.29	0.00289	0.772	0.193
10	12.7	0.0380	5.23	1.430
15	27.8	0.138	13.2	3.933
20	43.47	0.3165	23.30	7.475
25	58.37	0.5714	34.62	11.76
30	72.18	0.8984	46.50	16.55
35	84.19	1.290	58.56	21.69
40	94.55	1.737	70.49	27.05
45	104.9	2.236	82.21	32.53
50	117.6	2.791	93.89	38.08
52.9	122.6	3.139	100.7	41.32
Crystal II				
52.9	122.6	3.271	103.2	41.32
60	128.1	4.165	119.0	49.60
70	135.0	5.481	139.3	60.99
80	140.7	6.860	157.7	71.94
90	146.2	8.294	174.6	82.42
100	152.1	9.785	190.3	92.43
110	158.4	11.34	205.1	102.0
120	165.2	12.96	219.1	111.2
130	172.6	14.64	232.6	120.0
140	181.3	16.41	245.7	128.5
150	192.3	18.28	258.6	136.8
160	206.9	20.27	271.5	144.8
170	226.7	22.43	284.6	152.6
Crystal I				
180	169.3	24.34	295.5	160.3
190	169.8	26.04	304.7	167.6
200	171.1	27.74	313.4	174.7
210	172.8	29.46	321.8	181.5
220	174.8	31.20	329.9	188.1
230	177.0	32.96	337.7	194.4
240	179.2	34.74	345.3	200.5
250	181.3	36.54	352.6	206.5
260	183.9	38.37	359.8	212.2
270	190.4	40.23	366.8	217.9
280	186.4	42.14	373.8	223.3
290	186.9	44.01	380.3	228.6
298.15	187.6	45.53	385.5	232.8
300	187.8	45.88	386.7	233.8
310	188.9	47.76	392.9	238.8
320	190.2	49.66	398.9	243.7
330	191.5	51.56	404.7	248.5
340	192.8	53.49	410.5	253.2
345	193.3	54.45	413.3	255.5

## Data Availability

Data are contained within the article.

## Supplementary Materials

The following are available online at https://www.mdpi.com/article/10.3390/e24020145/s1. Figure S1: Molar heat capacities of methylammonium lead iodide (MAPI) over the range from 5 to 357 K.
