# Supplementary material for: Thermodynamic Study of Formamidinium Lead Iodide (CH5N2PbI3) from 5 to 357 K
Source: Entropy (Basel). 2022 Jan 18;24(2):145. doi: 10.3390/e24020145 (PMC8871434; doi:10.3390/e24020145)
Supplement: Supplementary file 1 [file entropy-24-00145-s001.zip › entropy-1537793-supplementary.pdf]

entropy

*Supplementary Material*

# Thermodynamic study of formamidinium lead iodide ($CH_5N_2PbI_3$) from 5 to 357 K

**Andrea Ciccioli [1], Alessandro Latini [1], Alessio Luongo [1], Natalia N. Smirnova [2], Alexey V. Markin [2,*] and Stefano Vecchio Ciprioti [3,*]**

1 Department of Chemistry, Sapienza University of Rome, P.le A. Moro 5, Building CU014, I-00185 Rome,
2 National Research Lobachevsky State University of Nizhny Novgorod, 23/5 Gagarin Av., 603950 Nizhny Novgorod, Russia;
3 Department of Basic and Applied Science for Engineering (S.B.A.I.), Sapienza University of Rome, Via del Castro Laurenziano 7, Building RM017, I-00161 Rome, Italy; stefano.vecchio@uniroma1.it
* Correspondence: markin@calorimetry-center.ru (A.V.M.); stefano.vecchio@uniroma1.it (S.V.C.)

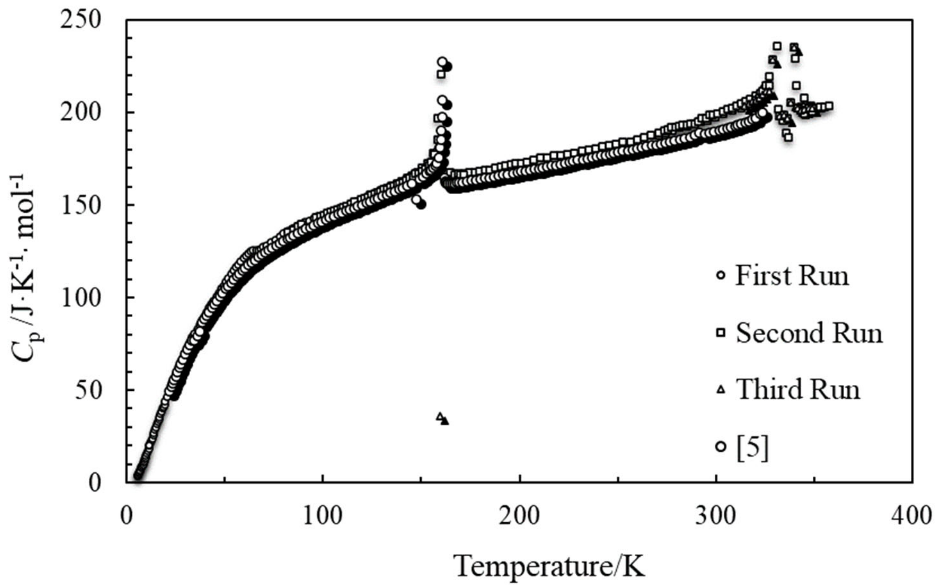


**Figure S1.** Molar heat capacities of methylammonium lead iodide (MAPI) over the range from 5 to 357 K
